# Management of recurrent hemoptysis: a single-center experience

**DOI:** 10.55730/1300-0144.5534

**Published:** 2022-07-09

**Authors:** Seda Tural ÖNÜR, Sedat ALTIN, Fatma Tokgöz AKYIL, Kaan KARA, Sinem Nedime SÖKÜCÜ, Cengiz ÖZDEMİR, Mehmet Akif ÖZGÜL, Muzaffer METİN, Levent CANSEVER, Aysun ÖLÇMEN, Özgür KILIÇKESMEZ

**Affiliations:** 1 Department of Pulmonology, Yedikule Chest Diseases and Thoracic Surgery Education and Research Hospital, University of Health Sciences, İstanbul, Turkey; 2 Department of Thoracic Surgery, Yedikule Chest Diseases and Thoracic Surgery Education and Research Hospital, University of Health Sciences, Iİstanbul, Turkey; 3 Department of Interventional Radiodiagnostic, İstanbul Education and Research Hospital, University of Health Sciences, Iİstanbul, Turkey

**Keywords:** Hemoptysis, bronchoscopy, embolization, surgery, rigid

## Abstract

**Background/aim:**

A successful planning methodology for patients with hemoptysis promises overall improvement in patient care. Conducted in a reference center for chest diseases, the present study aims to analyze characteristics and predictors of interventional methods in patients with recurrent hemoptysis.

**Materials and methods:**

The present study is a single-center, retrospective observational study. Between 2015 and 2018, 5973 patients with follow-up data until 2021 requiring more than one hospitalization due to recurrent hemoptysis were investigated. Patient characteristics, the amount of hemoptysis, baseline admission parameters, interventional procedures of bronchial artery embolization (BAE), fiberoptic bronchoscopy, rigid bronchoscopy, and surgical resections applied were analyzed according to number of hospitalizations and outcome.

**Results:**

Hospital admission numbers were higher in patients with sequela of tuberculosis, bronchiectasis and lung cancer. While lung cancer was the most frequent underlying reason in recurrent admissions, it was determined that as the amount of bleeding increased, the number of admissions also increased to the hospital, and BAE and rigid bronchoscopy were performed more frequently in the groups with less frequent admissions. There was no statistically significance between the amount of bleeding, and the interventional procedure alone or in combination with another procedure (p > 0.05).

**Conclusion:**

In conclusion, patients with certain diseases may experience frequent hospital admissions due to hemoptysis. Recurrent admissions may get better results with BAE and rigid bronchoscopy. We think that these procedures should be preferred in the foreground of suitable patient selection in line with available facilities and experience.

## 1. Introduction

Hemoptysis is defined as mixed-blood or exclusively blood expectoration from lower respiratory tracts [1,2]. It is encountered frequently in medical practice as it overlaps with many underlying diseases. Hemoptysis may be life-threatening for about 5%–15% of the cases [3,4]. Massive hemoptysis is defined as 100–1000 mL or 300–600 mL bleeding amount accumulated within 24 h and requires immediate management [5,7]. Since the management of hemoptysis requires simultaneous use of different methods in both diagnosis and treatment, multidisciplinary collaboration is vital. After the hemodynamic stabilization is established, conservative interventional methods of urgent surgical procedure, bronchial artery embolization (BAE), and rigid bronchoscopy should be considered [8,9].

Although the surgery option is perceived as parenchyma-protective and thus favored in the early phases, urgent surgical interventions in hemoptysis corresponds to a 40% higher rate of mortality compared to the elective procedures [10]. Either BAE is not suitable or in patients with recurrent hemoptysis, a 48-h waiting period is recommended with lower mortality compared to opting for an early surgery. BAE has a success rate of 80%–99% despite a 12%–17% recurrence rate [9,11,12].

Fiberoptic bronchoscopy visualizing bronchi can be utilized to localize the site of bleeding in massive hemoptysis in 73%–93% of the cases, whereas localization success declines in mild bleeding [13–16]. Timing and speed of the intervention is critical in this phase. Rigid bronchoscopy, on the other hand, with a clear vision and respiratory safety, provides bleeding control and respiratory hygiene. To reach lower respiratory tract, fiberoptic bronchoscopy in conjunction with the rigid bronchoscopy allows complete and clear visualization [17]. Effective management of hemoptysis in experienced centers may decrease recurrence rates.

Performed in a reference center with high bed capacity, the aim of this study is to evaluate the success rates of alternative interventional methods in patients with recurrent hemoptysis.

## 2. Patients and methods

The present study is a single-center and retrospective, observational study. The data of the patients who visited emergency department (ED) with hemoptysis between January 2015 and January 2018, with a follow-up data until 2021, were retrieved from the hospital database system.

Among 22,212 patients with at least one ED visit with hemoptysis, 5973 patients with at least one hospitalization were included in the study (Figure). The study was conducted in accordance with the principles of the Declaration of Helsinki and the study protocol was approved by the Ethics Committee of Health Sciences University İstanbul Yedikule Chest Diseases Training and Research Hospital (No: 2020-40)

### 2.1. Organization of the ED

In ED, after hemodynamic stabilization, adrenalin nebulization is used; in localized cases, cold compress and intravenous tranexamic acid are administered. When needed, fresh plasma and thrombocyte infusion are used in patients with coagulation disorder. When needed, interventional procedures such as rigid bronchoscopy and bronchial artery embolization are performed within the first 48 h in patients with hemoptysis. For BAE, cases are referred to a single-center radio-diagnostic unit (Ö.K.). Transfemoral way is used to access the routine bronchial circulation. Polyvinyl alcohol of 300–500 nm is used in embolization.

### 2.2. Recorded parameters

Patients’ demographics, comorbid diseases, date of hospital admission, duration of hospitalization, invasive procedures throughout the hospitalization were noted. The invasive procedures were flexible or rigid bronchoscopy, BAE, and surgical procedures which were decided by the expert team. The following values at admission were recorded as baseline parameters: complete blood count (CBC), platelet count (Plt), liver function tests (aspartate aminotransferase (AST) and alanine aminotransferase (ALT)), and renal function tests. Bleeding amounts of all patients at ED visit were classified as minor, moderate, or massive.

During follow-up, all interventional methods of either flexible or rigid bronchoscopy, BAE, and surgical procedures applied for hemoptysis were investigated.

All cases’ survival statuses were checked via National Death Registration. Censored via death status, patients’ readmissions with recurrent hemoptysis were recorded until January 2021. Baseline parameters and invasive procedures according to recurrence of hemoptysis were analyzed.

### 2.3. Definitions

In present study, the accepted classification for massive hemoptysis is the bleeding amount of 100–1000 mL or 300–600 mL within 24 h [5–7]. Severe hemoptysis is defined as a bleeding amount of 150 mL within 12 h [8]. Moderate hemoptysis: bleeding amount of 100–300 mL/day and mild hemoptysis is described as bleeding less than 100 mL/day [10].

### 2.4. Study design

Recorded parameters at hospital admission with hemoptysis and invasive procedures undertaken were investigated. The relationship between recorded parameters and patients’ readmissions were analyzed.

Additionally, the relationship between the procedures undertaken and mortality was investigated.

### 2.5 Statistical analysis

SPSS 21.0 (IBM, Armonk, NY, USA) program was used for statistical analysis. Descriptive statistics are presented as mean, standard deviation, frequency, and percentages. Recurrence rates are shown as categorical variables; comparisons were made via chi-squared test. Likewise, the chi-squared test was used to compare the bleeding amount and the interventional procedures. Bonferroni correction was made for pairwise comparisons of the variables that were significant as a result of the chi-squared test. The compliance of the follow-up duration with the normal distribution was evaluated in line with the Shapiro–Wilk test. The Mann–Whitney U test was used to compare the procedures performed in the two groups, since it did not meet the normal distribution assumption. The Kruskal–Wallis H test was used to compare laboratory parameters with the number of admission. Dunn’s test was used in post hoc analysis. The p < 0.05 value was accepted as significant.

## 3. Results

Of all the 5973 patients, the mean age was 50 ± 16 (14–91) and 4249 (71%) were male. Current smoking rate was 37% while 55% were never-smoker. During follow-up, hemoptysis recurred twice in 2644, thrice in 1543, and four times or more in 1786 patients. Age (49 ± 16; 50 ± 16; 49 ± 16; p = 0.959), sex, and smoking status did not affect the frequency of recurrence (p = 0.296, p = 0.913, respectively). Among recurrent cases, lung cancer (n = 373), bronchiectasis (n = 248), tuberculosis (TB) sequelae (n = 166), active TB (n = 125), and pulmonary thromboembolism (n = 59) coexisted. Comorbid diseases such as active tuberculosis (TB), history of TB, bronchiectasis, and lung cancer amplified recurrence (p < 0.001, for all) (Table 1).

The interventions for patients with lung cancer were BAE in 40%, (n = 150), rigid bronchoscopy in 24% (n = 90), rigid bronchoscopy + BAE in 11% (n = 42), and BAE + rigid bronchoscopy + surgical resection in 2% (n = 8). In bronchiectasis, 40% (n = 100) underwent BAE, 30% (n = 74) rigid bronchoscopy, 14% (n = 35) rigid bronchoscopy + BAE, and 3% (n = 8) underwent rigid bronchoscopy + BAE + surgical resection.

As for laboratory parameters, baseline lower hemoglobin and hematocrit values were correlated with higher number of admissions which may also be related to blood loss due to hemoptysis (p < 0.001, for all). The other total blood count and biochemistry values had no impact on recurrence (Table 2).

A statistically significant correlation was found between the admission numbers and BAE, number of BAEs, and rigid bronchoscopy in the study (p < 0.001, p = 0.048 and p < 0.001, respectively). Table 3 shows the relationship between admission numbers and interventional methods.

A statistically significant correlation was found between the amount of bleeding and the operation, type of operation, BAE, and number of BAEs in the study (p = 0.046, p = 0.040, p = 0.003, and p = 0.034, respectively) (Table 4). No statistical significance was observed in terms of performing a distinct interventional procedure or a combination of interventional procedures with recurrence rates and bleeding amount (p > 0.05) (Table 5,6).

Overall follow-up duration was 1260 ± 905 days in all patients whereas lung cancer patients were followed up for 660 ± 354 days. Patients undergoing BAE were followed up longer compared to patients undergoing BAE + rigid bronchoscopy and BAE + surgical resection (p = 0.011 and p < 0.001, respectively). Sole rigid bronchoscopy group had a longer follow-up period compared to the rigid bronchoscopy + operation group (p = 0.022). There was no statistical difference in the follow-up period of the group undergoing rigid bronchoscopy and rigid bronchoscopy + BAE (p > 0.05) (Table 7).

## 4. Discussion

We believe that our study contributes to the existing literature by showing that the combination of BAE and rigid bronchoscopy has the potential to moderate hospital admission episodes of the patients compared to the separate use of each modality. We found that as bleeding increases, bundling of different interventional methods becomes more common. BAE and rigid bronchoscopy were the most frequently combined methods applied to our patients who also happened to have the longest follow-up terms.

In daily practice, hemoptysis is encountered as simply symptoms of various diseases and a significant number of the hemoptysis cases may be life-threatening [1–3]. Management of hemoptysis in daily practice is challenging due to the following reasons: the fact that many diseases can cause hemoptysis, only a few centers/facilities are experienced in managing it, and even fewer hospitals/centers are qualified enough to intervene massive hemoptysis competently. This study, conducted in a specialized and reference center with a robust set of cases and an immensely experienced team of doctors, can cast light on customized management methods according to the patients’ needs through comparing different modalities.

Abdulmalak et al. analyzed patients with hemoptysis with a mean age of 62 and female:male ratio as 2:1 [18]. In parallel, the average age at admission and female to male ratios are similar in our study. Smoking history was not found as meaningfully related to hemoptysis in Adelman et al.’s study where 71% of the patients were found to have smoking history [19]. Similarly, 45% of our cases were ever-smoker and no significant relationship was found between bleeding and smoking history.

In European countries, an underlying cause is reported to be identifiable in 50% of hemoptysis patients, and this cause is mostly lung-related [18]. In other studies, the underlying cause of hemoptysis could not be revealed in 5%–34% of patients admitted with hemoptysis [20].

Lung diseases are categorized generally as (although subcategories may differ slightly) the following: air-borne infections, bronchial carcinomas/metastases, bronchiectasis/cystic fibrosis, pulmonary edema/mitral stenosis, and TB [18]. Massive hemoptysis is most frequently caused by TB, bronchiectasis, mycetoma, and lung cancer, respectively; TB is especially the most prevalent reason in endemic regions [3]. In present study, the most encountered causes in recurrence and frequent admissions were lung cancer followed by bronchiectasis, TB sequela, and active TB. This departure from literature may be explained by our hospital’s being a reference hospital and thus the referral of a significant proportion of malignant cases to our center. As stated above, the immersive hemoptysis experience of our hospital is also a reason for our routine admission of these patients from various other hospitals. Furthermore, because our study purposefully covers only the patients who had to be hospitalized for treatment and who had been admitted to the hospital at least twice, we automatically excluded the patients whose hemoptysis is rather minimal. While the majority of hemoptysis cases comprise light bleeding, 5%–15% are reported as massive bleeding [3–4]. In line with the literature, 3.8% of our patients were bleeding massively.

Flexible bronchoscopy, used for verification of bleeding, bleeding localization, endobronchial blocker placement, selective intubation, and clot extraction, is the most frequently used interventional method for diagnostic and therapeutic purposes in hemoptysis [3,20,21]. It can detect the focus of bleeding with 73%–93% certainty [13–22]. It is not only important for better examination of subsegments and detection of bleeding focus but also it can control bleeding in acute cases as bronchoscopy provides bufferization. Other tools for bleeding control are medical processes aiming the bleeding focus through as epinephrine, tranexamic acid, and cold saline [13–23]. In our study, in parallel to the literature, 80% of the patients underwent flexible bronchoscopy. It was specifically opted for in mild (51%) and moderate (45%) cases.

Rigid bronchoscopy may be preferred over flexible bronchoscopy in the presence of following conditions: the ability to simultaneously provide airway safety and intervention, allowing isolation of the primary bronchus, providing a larger channel for intervention and thus enabling rapid removal of sizeable clots, allowing additional intervention of thermal ablation or cauterization and also allowing the use of additional tools such as bronchial balloon or tamponades [23–24]. In line with the literature, in our study, it is observed that rigid bronchoscopy was prevalent in mild and moderate cases while in massive bleeding cases, compared to other treatment modalities, it was the first and most preferred interventional method.

In the analysis by Panda et al. where 22 studies were assessed, the success rate of BAE was detected as 70%–90% and the recurrence rate of hemoptysis in BAE-applied patients who also had aspergillum, TB, and bronchiectasis rose to a value as high as 58% [9,25]. In our study, %43.6 underwent BAE procedure and those patients with a high amount of bleeding and thus with frequent admissions to hospital were especially referred to undergo BAE. It was the most preferred interventional method to be followed by rigid bronchoscopy in our study.

Surgical resections are increasingly less preferred in recent years due to the following reasons: the rather exclusive application of pulmonary resection under emergency conditions in patients not otherwise responding to medical treatment, pneumonectomy being favored as the radical surgical method, and postoperation complications [26]. Similarly, in our study, surgical resections was performed solely in critical cases of massive life-threatening bleeding when the source of bleeding could be detected and the cause of bleeding could be quenched via interventional methods [27]. In the multicenter study by Lee et al., less than 1% of hemoptysis patients who were defined as emergency cases underwent surgical operation [28]. We observed in our study that 22.5% of the patients underwent surgical resection, of which 90% were wedge resection. As given above, because our center accepts mostly emergency cases and it is a primary referral center for cases such as lung cancer, bronchiectasis, and mycetoma which have specific causes requiring surgical operation, the higher rate of resections compared to that in the literature is not surprising. It should also be clarified that of these surgical methods, parenchymal protective surgery was the main one.

The main limitation of our study is that the retrospective design could not eliminate the impact of compounding factors such as medical history and previous medical treatment on the intervention success or failure. Also, our hospital’s being a reference center may have an inevitable impact on the case profile, thus the results. Because our patients were rather severe cases and referred from other various centers, the influence of their very initial hemoptysis on the intervention success could not be calculated. Finally, a detailed analysis of different diseases could not be given in this study. We believe that a prospective design where bleeding amount is set beforehand would guide for better practice and testing the retrospective findings. On the other hand, the high number of patients who have been followed up in a specific reference experienced center, giving an overall result of interventional methods is the strength of this study. In conclusion, hemoptysis patients comprise a medical emergency requiring frequent emergency room visits and hospitalization. This inevitably makes this patient group very important for management prospects and cost-effectiveness purposes. The extant literature involves studies with rather small number of cases where treatment modalities’ comparison is not sufficiently satisfactory. Our study is contributory in this respect with its set of severe patients, indicating that the combined use of BAE and rigid bronchoscopy could be considered for successful hemoptysis control.

## Figures and Tables

**Figure:**
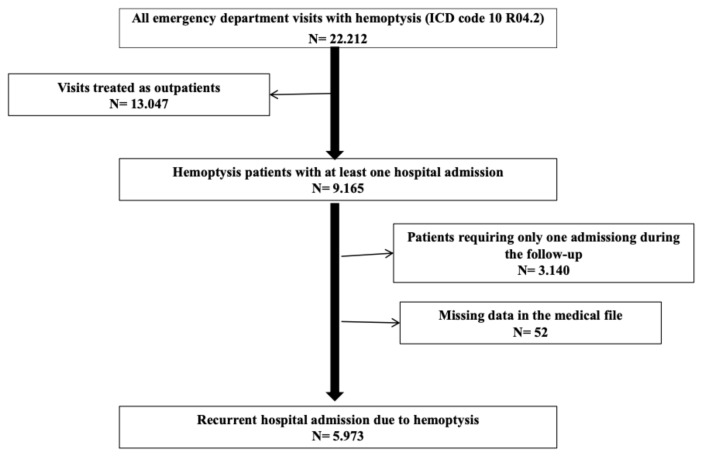
Flowchart of patient inclusion.

**Table 1:** Comparison of number of admissions and clinical parameters.

	Total	Twice	Thrice	≥ four times	p
Sex					
**Female**	1724 (28.86 )	790 (29.88)	430 (27.87)	504 (28.22)	0.296
**Male**	4249 (71.14 )	1854 (70.12)	1113 (72.13)	1282 (71.78)	
Smoking status					
**Current smoker**	2189 (36.85 )	985 (37.44)	559 (36.35)	645 (36.4)	0.913
**Never-smoker**	3249 (54.69 )	1428 (54.28)	850 (55.27)	971 (54.8)	
**Former smoker**	503 (8.47 )	218 (8.29)	129 (8.39)	156 (8.8)	
**TB**					
**No**	5848 (97.91 )	2582 (97.66)	1519 (98.44)	1747 (97.82)	0.216
**Yes**	125 (2.09 )	62 (2.34)	24 (1.56)	39 (2.18)	
**TB sequela**					
**No**	5807 (97.22 )	2599 (98.3)	1504 (97.47)	1704 (95.41)	**<0.001**
**Yes**	166 (2.78 )	45 (1.7)	39 (2.53)	82 (4.59)	
Bronchiectasis					
**No**	5725 (95.85 )	2557 (96.71)	1481 (95.98)	1687 (94.46)	**<0.001**
**Yes**	248 (4.15 )	87 (3.29)	62 (4.02)	99 (5.54)	
Lung cancer					
**No**	5600 (93.76 )	2498 (94.48)	1464 (94.88)	1638 (91.71)	**<0.001**
**Yes**	373 (6.24 )	146 (5.52)	79 (5.12)	148 (8.29)	
Pulmonary **embolism**/DVT					
**No**	5914 (99.01 )	2622 (99.17)	1535 (99.48)	1757 (98.38)	**0.003**
**Yes**	59 (0.99 )	22 (0.83)	8 (0.52)	29 (1.62)	

DVT: deep vein thrombosis, TB: tuberculosis. In paired comparisons; patients suffering from TB sequela experienced more frequent admissions than both twice and thrice. For bronchiectasis, four times admissions were more frequent than twice. Four times admissions were significantly higher than twice and thrice in lung cancer and in pulmonary emboli or deep vein thrombosis patients.

**Table 2:** Comparison of number of admissions and laboratory parameters.

	Total	Twice	Thrice	≥ four times	p
**Hemoglobin (g/dL)**	13.4 ± 2	13.5±2	13.6±1.9	13.3±2	**0.001**
Hematocrit (%)	40.1 ± 5.5	40.1±5.5	40.4±5.1	39.6±5.8	**0.001**
**Platelets**	258.1 ± 90.2	257.7 ± 87.6	255.54 ± 90.45	260.9 ± 93.6	0.322
**Creatinine (mg/dL)**	0.91 ± 0.76	0.88 ± 0.66	0.92 ± 0.81	0.93 ± 0.84	0.219
BUN **(U/L)**	34.5 ± 16.1	34.6 ± 16.6	34.6 ± 16.9	34.1 ± 14.4	0.729
ALT **(U/L)**	21.8 ± 22	22.4 ± 23.7	21.6 ± 18.4	21.1 ± 22.1	0.359
AST **(U/L)**	24.3 ± 17.4	24.7 ± 19.7	24.7 ± 17.8	23.3 ± 13.1	0.095
GGT **(U/L)**	40.1 ± 57.4	40.7 ± 53.9	37.6 ± 53.1	41.1 ± 64.	0.587
CRP **(mg/L)**	33. ± 55.5	35.1 ± 58.9	30.9 ± 53.6	31.8 ± 52.	0.138
**Albumin ** **(g/dL)**	4 ± 0.5	4 ± 0.6	4 ± 0.5	4 ± 0.5	0.144

ALT: alanine transaminase, AST: aspartate transaminase; BUN: blood urea nitrogen, CRP: C-reactive protein, GGT: gamma-glutamyl transpeptidase. In paired comparisons, lower hemoglobin and lower hematocrit levels were correlated with more frequent four times admissions.

**Table 3:** Comparison of number of admissions and interventional methods.

	Total	Twice	Thrice	≥ four times	p
Surgical operation					
**No**	4628 (77.5)	2065 (78.1)	1180 (76.5)	1383 (77.4)	0.477
**Yes**	1345 (22.5)	579 (21.9)	363 (23.5)	403 (22.6)	
Operation					
**Wedge**	1209 (20.2)	520 (19.7)	320 (20.7)	369 (20.7)	0.676
**Lobectomy**	117 (2.0)	51 (1.9)	37 (2.4)	29 (1.6)	
**Pneumonectomy**	19 (0.3)	8 (0.3)	6 (0.4)	5 (0.3)	
BAE					
**No**	3371 (56.4)	1344 (50.8)	968 (62.7)	1059 (59.3)	**<0.001**
**Yes**	2602 (43.6)	1300 (49.2)	575 (37.3)	727 (40.7)	
Total number of BAE					
**1**	2323 (89.3)	1157 (89.0)	525 (91.3)	641 (88.2)	**0.048**
**2**	186 (7.1)	88 (6.8)	41 (7.1)	57 (7.8)	
**3**	93 (3.6)	55 (4.2)	9 (1.6)	29 (4.0)	
Rigid bronchoscopy					
**No**	3475 (58.2)	1414 (53.5)	978 (63.4)	1083 (60.6)	**<0.001**
**Yes**	2498 (41.8)	1230 (46.5)	565 (36.6)	703 (39.4)	
Flexible Bronchoscopy					
**No**	1144 (19.2)	519 (19.6)	280 (18.1)	345 (19.3)	0.490
**Yes**	4829 (80.8)	2125 (80.4)	1263 (81.9)	1441 (80.7)	
Total number of flexible bronchoscopy					
**1**	93 (1.9)	43 (2.0)	23 (1.8)	27 (1.9)	0.395
**2**	1298 (26.9)	548 (25.8)	334 (26.4)	416 (28.9)	
**3**	1939 (40.1)	884 (41.6)	506 (40.1)	549 (38.1)	
**4**	1500 (31.1)	651 (30.6)	400 (31.7)	449 (31.2)	
Amount of bleeding					
Mild	3044 (51.0)	1471 (55.6)	764 (49.5)	809 (45.3)	**<0.001**
Moderate	2701 (45.2)	1061 (40.2)	730 (47.3)	910 (51.0)	
Massive	228 (3.8)	112 (4.2)	49 (3.2)	67 (3.9)	

BAE: bronchial artery embolization. In paired comparisons, four times admissions were significantly less frequent than twice and thrice in patients undergoing either BAE or rigid bronchoscopy. The amount of bleeding was mostly mild in twice admissions.

**Table 4:** Comparison of interventional methods according to the amount of bleeding.

	Total	Mild	Moderate	Massive	p
Operation					
**No**	4628 (77.5)	2352 (77.3)	2084 (77.2)	192 (84.2)	**0.046**
**Yes**	1345 (22.5)	692 (22.7)	617 (22.8)	36 (15.8)	
Operation **type**					
**Wedge resection**	1209 (20.2)	631 (20.7)	544 (20.1)	34 (14.9)	**0.040**
**Lobectomy**	117 (2)	51 (1.7)	65 (2.4)	1 (0.4)	
**Pneumonectomy**	19 (0.3)	10 (0.3)	8 (0.3)	1 (0.4)	
BAE					
**No**	3371 (56.4)	1769 (58.1)	1462 (54.1)	140 (61.4)	**0.003**
**Yes**	2602 (43.6)	1275 (41.9)	1239 (45.9)	88 (38.6)	
Total number of BAE					
**1**	2323 (89.3)	1159 (90.9)	1082 (87.3)	82 (93.2)	**0.034**
**2**	186 (7.1)	79 (6.2)	104 (8.4)	3 (3.4)	
**3**	93 (3.6)	37 (2.9)	53 (4.3)	3 (3.4)	
Rigid bronchoscopy					
**No**	3475 (58.2)	1798 (59.1)	1545 (57.2)	132 (57.9)	0.358
**Yes**	2498 (41.8)	1246 (40.9)	1156 (42.8)	96 (42.1)	
Flexible bronchoscopy					
**No**	1144 (19.2)	581 (19.1)	517 (19.1)	46 (20.2)	0.922
**Yes**	4829 (80.8)	2463 (80.9)	2184 (80.9)	182 (79.8)	
Total number of flexible bronchoscopy					
**1**	93 (1.9)	48 (1.9)	44 (2)	1 (0.5)	0.640
**2**	1298 (26.9)	669 (27.2)	581 (26.6)	48 (26.4)	
**3**	1939 (40.1)	979 (39.7)	877 (40.2)	83 (45.6)	
**4**	1500 (31.1)	768 (31.2)	682 (31.2)	50 (27.5)	

BAE: bronchial artery embolization. In paired comparisons, in patients undergoing an operational procedure, massive bleeding was less frequent than mild and moderate, BAE was less frequent in mild hemoptysis than moderate amount. Total number of BAE was correlated with moderate and mild bleeding amount.

**Table 5:** Comparison of number of admissions and interventional methods.

	Total	Twice	Thrice	≥ four times	p
BAE	249 (21.4)	134 (23.3)	52 (19.6)	63 (19.5)	0.305
BAE + Rigid **bronchoscopy**	915 (78.6)	442 (76.7)	213 (80.4)	260 (80.5)	
BAE	249 (30.1)	134 (32)	52 (28.3)	63 (28.1)	0.493
BAE + operation	578 (69.9)	285 (68)	132 (71.7)	161 (71.9)	
Rigid	246 (21.2)	121 (21.5)	51 (19.3)	74 (22.2)	0.680
Rigid + BAE	915 (78.8)	442 (78.5)	213 (80.7)	260 (77.8)	
Rigid	246 (29.9)	121 (31.1)	51 (27.7)	74 (29.5)	0.702
Rigid + operation	578 (70.1)	268 (68.9)	133 (72.3)	177 (70.5)	

BAE: bronchial artery embolization.

**Table 6:** Comparison of amount of bleeding and interventional methods.

	Total	Mild	Moderate	Massive	p
BAE	249 (21.4)	126 (21.5)	114 (21.3)	9 (20)	0.970
BAE + rigid **bronchoscopy**	915 (78.6)	459 (78.5)	420 (78.7)	36 (80)	
BAE	249 (30.1)	126 (30.7)	114 (28.9)	9 (42.9)	0.373
BAE + operation	578 (69.9)	285 (69.3)	281 (71.1)	12 (57.1)	
Rigid	246 (21.2)	121 (20.9)	116 (21.6)	9 (20)	0.932
Rigid + BAE	915 (78.8)	459 (79.1)	420 (78.4)	36 (80)	
Rigid	246 (29.9)	121 (29.3)	116 (29.7)	9 (42.9)	0.415
Rigid + operation	578 (70.1)	292 (70.7)	274 (70.3)	12 (57.1)	

BAE: bronchial artery embolization.

**Table 7:** Comparison of follow-up duration (days) according to interventional methods.

	N	Mean ± SD	P
BAE	249	1416.9 ± 650.6	**0.011**
BAE + Rigid **bronchoscopy**	915	1295.7 ± 664.8	
BAE	249	1416.9 ± 650.6	**<0.001**
BAE + operation	578	1234 ± 672.2	
Rigid **bronchoscopy**	246	1382.8 ± 659.5	0.068
Rigid **bronchoscopy** + BAE	915	1295.7 ± 664.8	
Rigid **bronchoscopy**	246	1382.8 ± 659.5	**0.022**
Rigid **bronchoscopy** + operation	578	1266.4 ± 670	

BAE: bronchial artery embolization.
